# Unveiling the effects of *Rosa canina* oligosaccharide liposome on neuropathic pain and motor dysfunction following spinal cord injury in rats: relevance to its antioxidative effects

**DOI:** 10.3389/fphar.2025.1533025

**Published:** 2025-02-14

**Authors:** Yasaman Ahmadpour, Gholamreza Bahrami, Elham Arkan, Fatemeh Abbaszadeh, Faranak Aghaz, Sajad Fakhri, Javier Echeverría

**Affiliations:** ^1^ Student Research Committee, Kermanshah University of Medical Sciences, Kermanshah, Iran; ^2^ Medical Biology Research Center, Health Technology Institute, Kermanshah University of Medical Sciences, Kermanshah, Iran; ^3^ Nano Drug Delivery Research Center, Health Technology Institute, Kermanshah University of Medical Sciences, Kermanshah, Iran; ^4^ Neurobiology Research Center, Institute of Neuroscience and Cognition, Shahid Beheshti University of Medical Sciences, Tehran, Iran; ^5^ Pharmaceutical Sciences Research Center, Health Institute, Kermanshah University of Medical Sciences, Kermanshah, Iran; ^6^ Departamento de Ciencias del Ambiente, Facultad de Química y Biología, Universidad de Santiago de Chile, Santiago, Chile

**Keywords:** spinal cord injury, *Rosa canina*, oligosaccharide, nanoliposomes, oxidative stress, neuropathic pain, motor function

## Abstract

**Background:**

Spinal cord injury (SCI) is a leading cause of sensorimotor disorders, impacting millions of people globally. The absence of effective treatments and the side effects of existing medications highlight the need for innovative research into new therapeutic compounds.

**Purpose:**

Given the critical role of oxidative stress in the development of SCI and the antioxidant properties of oligosaccharides in other neurological disorders, this study focuses on the role of oxidative stress in SCI and explores the potential of a novel oligosaccharide nanoformulation derived from *Rosa canina* (Oligo-L).

**Materials and methods:**

Oligo-L was formulated using soy lecithin as the phospholipid and the characterization included size, zeta potential, morphology, and drug loading efficiency. Then 35 Wistar male rats were divided into five groups of Sham, SCI, and Oligo-L (10 μL intrathecal injection of 15, 30, and 45 mg/mL). An aneurysm clip was used to induce compression injury of the SCI and Oligo-L groups. Sensory-motor functions were evaluated weekly for 4 weeks using tests such as the BBB scale, inclined plane, acetone drop, hot plate, von Frey, and monitoring of weight changes. Additionally, oxidative stress markers and histological changes were examined to evaluate changes in nitrite, glutathione, catalase, and neuronal survival.

**Results and discussion:**

The findings indicated that Oligo-L treatment led to significant improvements in neuropathic pain, and motor function performance and weight of the animals from the first week post-SCI. Oligo-L also enhanced catalase and glutathione levels while reducing serum nitrite levels, contributing to neuronal preservation. Additionally, Oligo-L increased neuronal survival in the both ventral (motor neurons) and dorsal (sensory neurons) horns of the spinal cord.

**Conclusion:**

Overall, Oligo-L, characterized by its beneficial physicochemical properties, showed promising potential as a neuroprotective agent and facilitated the recovery of sensory and motor functions after SCI.

## 1 Introduction

Spinal cord injury (SCI) stands as a formidable public health challenge, afflicting millions of individuals worldwide and often resulting in debilitating sensorimotor disorders that significantly impair quality of life. The complexity of SCI is multifaceted, involving not only the immediate physical trauma to the spinal cord but also a cascade of biological responses that perpetuate damage and inhibit recovery (Cardile et al., 2024). Among the various biological mechanisms implicated in the pathophysiology of SCI, oxidative stress, characterized by an imbalance between reactive oxygen species (ROS) production and antioxidant defenses, has emerged as a critical factor that exacerbates neural damage and hinders recovery (Jia et al., 2012; Visavadiya et al., 2016). Nitric oxide (NO) is a significant reactive nitrogen species produced during oxidative stress (Ozcan and Ogun, 2015). Following SCI, there is an increased production of NO, which contributes to vasodilation. However, this rise in NO also nitrosylates the inflammatory pathways, leading to the formation of peroxynitrite, and various cell signaling messengers and oxidative damage to neurons, lipids, and DNA, which can further exacerbate neuronal damage (Conti et al., 2007; Xiong et al., 2007). Glutathione (GSH) is a critical non-enzymatic antioxidant that scavenges ROS, helping to maintain redox balance. It exists in two forms: reduced (GSH) and oxidized (GSSG). On the other hand, catalase is an enzyme that catalyzes the decomposition of hydrogen peroxide (H_2_O_2_), into water and oxygen. This reaction is crucial for mitigating oxidative stress (Brunelli et al., 2001; Vašková et al., 2023). After SCI, excess ROS can deplete GSH and overwhelm catalase, leading to increased oxidative stress (Jia et al., 2012).

Current treatment options for SCI are limited, despite advances in surgical techniques and rehabilitation therapies, the lack of effective and FDA-approved pharmacological interventions remains a pressing challenge. Existing drug treatments are often associated with undesirable side effects, which emphasizes the urgent need for innovative therapeutic strategies (Cristante et al., 2012).

One promising area of research focuses on the use of natural compounds known for their neuroprotective properties. *Rosa canina* L. [Rosaceae] (*R. canina*), also named dog rose, is a rich source of bioactive compounds, including oligosaccharides, which are known for their antioxidant and anti-inflammatory effects (Taneva et al., 2016). Recent studies have indicated that oligosaccharides derived from various plant sources can play a crucial role in modulating oxidative stress and promoting neuronal health (Vieira et al., 2020; Kang et al., 2022). Given the role of oxidative stress in the progression of SCI, the exploration of *R. canina* oligosaccharides as a potential therapeutic agent seems to be effective. We have previously characterized the aforementioned oligosaccharide used in the current study (Rahimi et al., 2020). In addition to their antioxidant properties, formulating oligosaccharides into liposomal carriers presents an innovative approach to enhancing their bioavailability and therapeutic efficacy (Deng et al., 2014; Jiang et al., 2023). Liposomes, spherical vesicles composed of lipid bilayers, can encapsulate bioactive compounds, protecting them from degradation and enabling targeted delivery to injured tissues. This method improves the stability of the oligosaccharides and facilitates their uptake by cells, thereby maximizing their potential benefits (Monteiro et al., 2014; Nsairat et al., 2022). The combination of *R. canina* oligosaccharides and liposomal technology could represent a groundbreaking strategy in the treatment of SCI.

This study aims to investigate the effects of a *R. canina* oligosaccharide liposomal formulation (Oligo-L) on sensory-motor function, following SCI in rats.

## 2 Materials and methods

### 2.1 Preparation of Oligo-L

To create the liposomes, briefly first 75 mg of soy lecithin dissolved in 2 mL of chloroform using an ultrasonication bath. The procedure resulting in a homogeneous suspension at 37°C with a rotation speed of 150 rpm for 15 min, which formed a thin lipid film layer once the solvent was fully removed. Next, the oligosaccharide extracted from *R. canina* (15 mg), was dissolved in 2 mL of distilled water at 37°C and added to the lipid film, resulting in a colored suspension of oligosaccharide-containing liposomes. To reduce the size of the multi-layered liposomes, an ultrasonication bath was used to remove excess layers. The synthesized liposomal suspension was then centrifuged at 15,000 rpm and 4°C for 20 min. After centrifuging, the resulting sediment (i.e., drug-loaded nanoliposomes) was freeze-dried for long-term storage (Manconi et al., 2016; Rahimi et al., 2020).

### 2.2 Determination of physical properties of liposomes

The hydrodynamic diameter and surface charge of particles were determined by the Dynamic Light Scattering (DLS) method utilizing the Zetasizer instrument (Nano-ZS, Malvern Instruments Ltd., Worcestershire, United Kingdom) at a controlled temperature of 25°C. In addition, a scanning electron microscope (SEM) (EM3200, KYKY Technology Co., China) operating at 25 kV, was utilized to examine the morphology and size of the synthesized liposomes (Baig et al., 2016). Moreover, the spectra of drug-free and drug-containing liposomal nanoparticles, as well as free oligosaccharides, were scanned using Fourier-Transform Infrared spectroscopy (FT-IR; IR Prestige-21, Shimadzu Co., Japan) in the range of 400–4,000 (cm^−1^) (Omwoyo and Moloto, 2019).

### 2.3 Measurement of drug loading

The encapsulated drug concentration in the liposome was ensured using liquid chromatography-mass spectrometry (LC-MS, Agilent Technologies, Germany). First, a standard calibration curve was prepared using various concentrations of the drug (1, 5, 10, and 15 mg/mL, straight line with r^2^ = 0.99). Next, the liposome solution was centrifuged at 18,200 g for 15 min, and the pellet of the un-entrapped drug was obtained after removing the supernatant. Then 1 mL of this medium was used for evaluating drug concentration via LC-MS. Finally, the drug loading (DL%) and entrapment efficiency (EE%) of the drug on liposome were calculated using the following formula:

$${\text{EE}}_{\%}=\left(W_{t}-W_{f}\right)/W_{t}\times 100$$

$${\text{DL}}_{\%}=\left(W_{t}-W_{f}\right)/W_{s}\times 100$$

Where W_t_, W_f_, and W_s_ represent the initial amount of the drug, the amount of free drug in the supernatant solution, and the total amount of the nanoliposomes, respectively (Chen et al., 2022).

### 2.4 Measurement of drug release rate

To quantify the drug release from the synthesized Oligo-L, 1 mL of a 30 mg/mL Oligo-L solution was dissolved in 25 mL of artificial cerebrospinal fluid. The artificial cerebrospinal fluid was made based on Düzlü et al. study (Düzlü et al., 2016). This mixture was incubated in a shaker incubator set at 80 rpm and maintained at 37°C. At specific time intervals, a 100 μL sample was collected from the solution and replaced with an equal amount of artificial cerebrospinal fluid to maintain consistent conditions. Each sample was then centrifuged at 14,000 rpm for 3 min, and the resulting supernatant was analyzed using high-performance liquid chromatography and a Diode Array Detector. An Agilent 1,200 series LC system was used with a column (150 × 4.6 mm), a quaternary delivery pump, a thermostated column compartment, and a degasser (Agilent Technologies, Germany) possessing a 20 μL sample loop Rheodyne 7725i manual injector valve (Cotati, CA, United States) (Rahimi et al., 2020). The data obtained were subsequently evaluated using a calibration curve (Abdoli et al., 2020).

### 2.5 Investigation of cytotoxicity

The MTT (3-[4,5-dimethylthiazol-2-yl]-2,5 diphenyl tetrazolium bromide) test was employed to assess cytotoxicity. Initially, the concentrations of 10, 20, 40, 80, 160, and 320 μg/mL of Oligo-L were prepared using phosphate buffer. Then, the pheochromocytoma cell lines were cultured in a 96-well plate and treated with these concentrations of Oligo-L. Following the treatment, the contents of the wells were completely removed, and the cells were washed with phosphate buffer. Next, 20 μL of yellow MTT solution was added to each of the wells, and the plate was incubated away from light for 4 h. Subsequently, 60 μL of DMSO solution was added to each well, and the plate was shaken for 10 min. Finally, the light absorption was measured at a wavelength of 490 nm using an ELISA reader (Abdoli et al., 2021).

### 2.6 Animal study

#### 2.6.1 Experimental animals

In total, 35 male Wistar rats, weighing between 230–260 g, were sourced from the animal facility at Kermanshah University of Medical Sciences (KUMS). The rats were accommodated in a controlled setting with a 12-h light-dark cycle, maintained at a temperature of 24°C, and provided with *ad libitum* access to food and water. The research was conducted in compliance with the protocols established by the KUMS’s animal care committee (IR.KUMS.REC.1399.083).

The rats were categorized into the following groups: Sham, SCI groups treated with drug-free liposome (as the vehicle), and three treatment groups (Olig-L) given 10 μL intrathecal (i.t.) doses of 15, 30, and 45 mg/mL of Oligo-L, respectively.

#### 2.6.2 Spinal cord injury

The rats were given an intraperitoneal (i.p.) injection of ketamine/xylazine (80/10 mg/kg) for anesthesia. After shaving, a 2 cm incision was made in the skin over the 8th and 9th thoracic vertebrae. Then, a vertebra fracture or laminectomy was performed using a rongeur (Fine Science Tools, United States). In case of bleeding, a subcutaneous (s.c.) injection of physiological saline was administered to replace lost blood. Following the laminectomy, an aneurysm clip was used to compress the spinal cord with 90 g of force for 1 minute, resulting in complete paralysis of the animal’s hind legs. The muscles and skin were then sutured using 0.3 threads. After surgery, the rats received s.c. injection of physiological saline (2 mL) and 40 mg/kg of intramuscular cefazolin to ensure rehydration and reduce the risk of infection, respectively. The animals’ bladders were manually emptied twice daily until the reflex returned (Fakhri et al., 2021; Bagheri Bavandpouri et al., 2024). Finally, 30 min post-SCI, rats were given i.t. doses of 10 μL of 15, 30, and 45 mg/mL Oligo-L or drug-free liposome.

#### 2.6.3 Behavioral test

All animals underwent sensory-motor behavioral testing and weighting before surgery (day 0) and then weekly post-surgery on days 7, 14, 21, and 28.

#### 2.6.4 Evaluation of neuropathic pain

The study utilized von Frey filaments to assess mechanical allodynia and sensitivity to contact stimuli. To acclimate the treated groups to the testing environment, they were placed in a test chamber 15 min before the experiment. Subsequently, von Frey filaments were applied with mild pressures of 10, 15, 26, 60, and 100 g between the 2nd and 3rd toes. Each filament was tested five times with 10–15 s intervals. If there was a positive response in at least 3 out of 5 repetitions, it was considered as the mechanical pain threshold (Fakhri et al., 2022).

The response of the rats to the thermal pain stimulus was evaluated by the hot plate device. Rats were placed in a cylindrical chamber on a hot plate at an adjustable temperature. To adapt and reduce the rats’ stress, before turning on the device, the animals were familiarized with the environment of the test plate. To perform the test, the rats were placed on a hot plate with a temperature of 52°C ± 2°C. The thermal pain threshold was considered as the time interval between placing the rat on the hot plate and the animal’s reaction to the pain stimulus in the form of jumping and paw licking. The time limit for this test was set at 60 s, and to confirm the test, each rat was placed on the plate 3 times at 5-min intervals. Then the obtained average was used for statistical analysis (Fakhri et al., 2019).

Cold allodynia in animals was evaluated by an acetone drop test. Briefly, 10 min after placing the animals in a special cage, 100 μL of acetone was sprayed on the rat’s soles from a distance of 2 cm. Sensitivity to cold was recorded by scoring the severity of the response (0 = no response, 1 = startle response without paw withdrawal, 2 = partial withdrawal, 3 = prolonged withdrawal, 4 = repeated withdrawal and licking) (Fakhri et al., 2018).

#### 2.6.5 Assessment of motor activity

The Basso-Beattie-Bresnahan (BBB) score and inclined test were used to evaluate locomotor function in animals. The rats’ movements including joint movement, weight support, stepping, coordination, and paw placement were monitored for 4 min and scored on a scale ranging from 0 for complete paralysis to 21 for normal movement (Fakhri et al., 2018).

For the inclined test, the wooden plate measuring 60 × 40 cm, adjustable from zero to 70°, was employed. The maximum angle at which each rat could maintain stability on the ramp for 5 s was documented. This test was conducted by two observers.

#### 2.6.6 Measurement of weight changes

The animals’ weights were recorded before surgery (day 0) and on the test days. Weight changes for each group were determined by subtracting the animal’s weight on days 7, 14, 21, and 28 from the weight on day zero (before surgery) (Fakhri et al., 2019).

#### 2.6.7 Biochemical assay

Following the behavioral evaluation on day 28, the rats were euthanized by CO2, and their serum samples were collected for biochemical analysis.

#### 2.6.8 Nitrite assay

The nitrite level was measured using the Greiss colorimetric method (Granger et al., 1995). Initially, serum samples were deproteinized with zinc sulfate and centrifuged at 10,000 rpm for 10 min. Subsequently, 100 μL of vanadium chloride solution (8 mg/mL) was combined with 100 μL of the supernatant to convert nitrate to nitrite. A Greiss solution containing 50 μL of 2% sulfanilamide and 50 μL of 0.1% ethylene diamine dihydrochloride was then added and incubated for 30 min at 37°C. The resulting color was measured at a wavelength of 540 nm.

#### 2.6.9 Catalase assay

Catalase activity was assessed using the Aebi method (Aebi, 1984). Each serum sample (20 μL) was dispensed into the plate wells, followed by the addition of 100 μL of 65 mM hydrogen peroxide. After incubation at 25°C, the reaction was stopped by adding 100 μL of 32.4 mM ammonium molybdate. Subsequently, the yellow complex formed was measured at a wavelength of 405 nm. Finally, the percentage difference in optical absorption between the SCI or Olig-L groups and the sham group was reported.

#### 2.6.10 GSH assay

The Ellman method was employed to quantify GSH levels (Ellman, 1959). 50 μL of phosphate buffer with a pH of 7.4 were added to each well. Subsequently, 40 μL of 5,5′-dithiobis (2-nitrobenzoic acid) (DTNB) was added to each well, resulting in the formation of a yellow-colored complex. The formed yellow complex was incubated for 10 min at 37°C and its absorbance was read at the wavelength of 412 nm. For both catalase and GSH, the percentage difference in optical absorbance between the treatment or SCI groups and the sham group was then calculated as;

$$\text{Concentration difference }\left(\%\right)=\left(\left(C_{\text{sham}}\;–\;C_{\text{sample}}\right)\;/\;C_{\text{sham}}\right)*100$$

#### 2.6.11 Histological examination

On the 28th day, rats underwent transcardial perfusion with approximately 200 mL of phosphate-buffered saline (pH 7.3) and the same volume of 4% paraformaldehyde. 1 cm of spinal cord tissue was collected from the site of injury, processed, and embedded in paraffin. Subsequently, 7-μm sections of paraffin-embedded tissue were prepared and stained with hematoxylin and eosin (H&E). Images were taken at ×40 magnification. The number of neurons in the dorsal and ventral horns was quantified using ImageJ software developed by the National Institutes of Health (NIH).

#### 2.6.12 Statistical analysis

The data were expressed as mean ± standard error of the mean (for analysis sections as S.E.M) and analyzed using GraphPad Prism software with one-way or two-way analysis of variance, followed by Tukey and Bonferroni post-tests. *p* < 0.05 was considered as statistical significance.

## 3 Results

### 3.1 Characteristics of nanoliposomes

**FIGURE 1:**
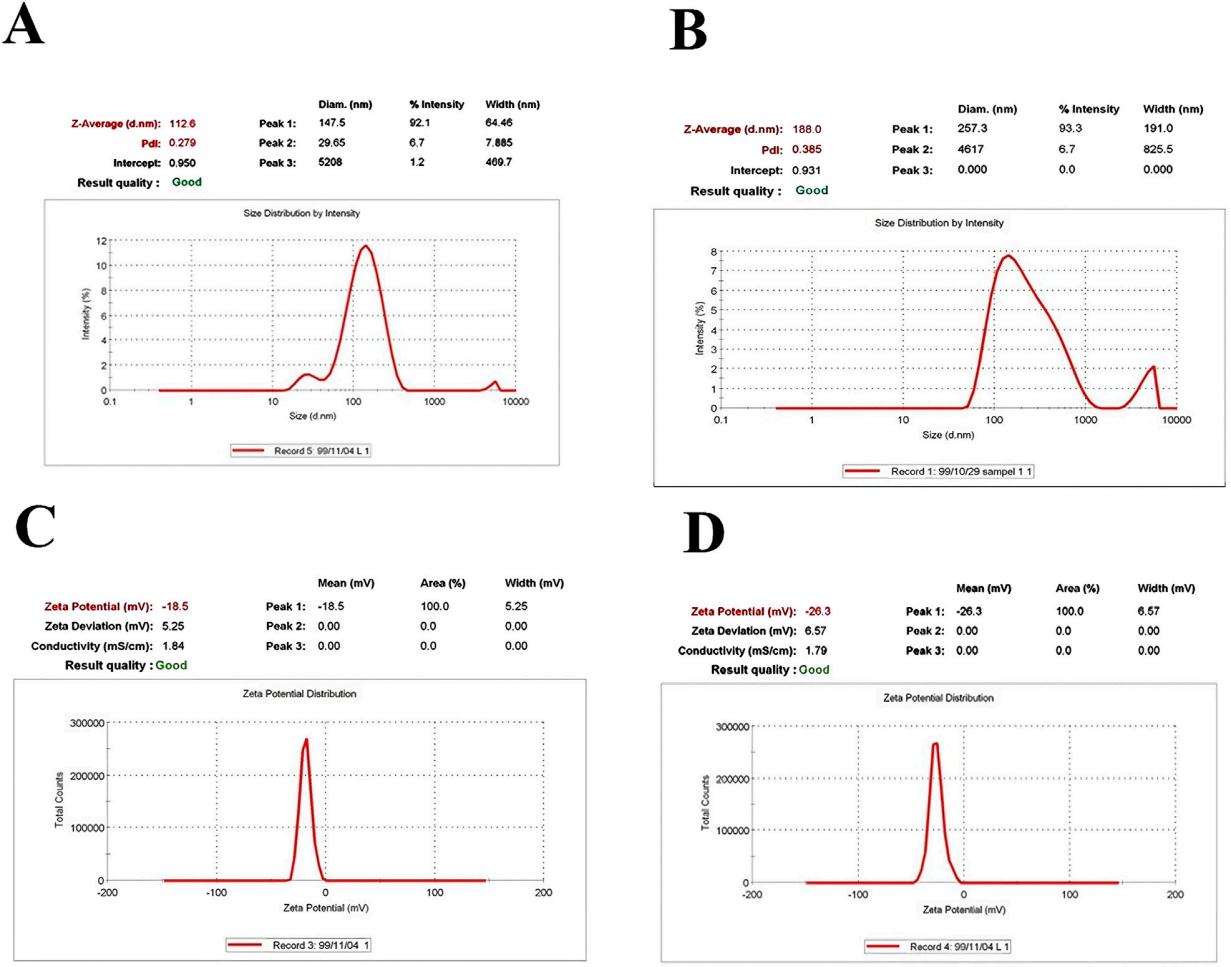
Physicochemical properties, including size and PDI of **(A)** drug-free nanoliposomes **(B)** nanoliposome-loaded drug; as well as zeta-potentials of **(C)** drug-free nanoliposomes **(D)** and nanoliposome-loaded drug.

The size distribution of particles was determined using DLS analysis. The DLS spectrum confirmed the size distribution and polydispersity index (PDI). The spectrum displayed an average size of 112.6 nm with a PDI of 0.279 for drug-free nanoliposomes (Figure 1A) and an average size of 188.0 nm with a PDI of 0.385 for nanoliposome-loaded drugs (Figure 1B). The zeta potential value was −18.5 mV for drug-free nanoliposomes (Figure 1C) and −26.3 for nanoliposome-containing drugs (Figure 1D).

**FIGURE 2:**
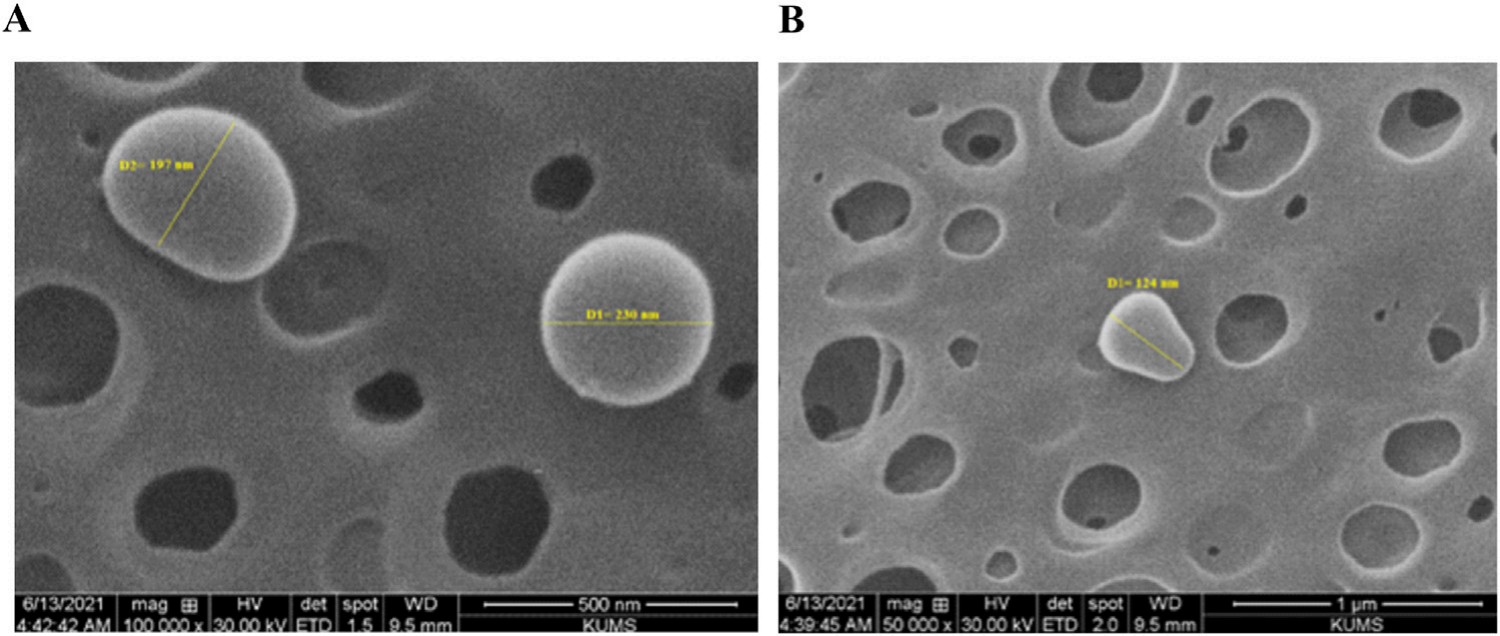
Scanning electron microscope images of oligosaccharide nanoliposome in magnifications of **(A)** 500 nm and **(B)** 1 µm.

**FIGURE 3:**
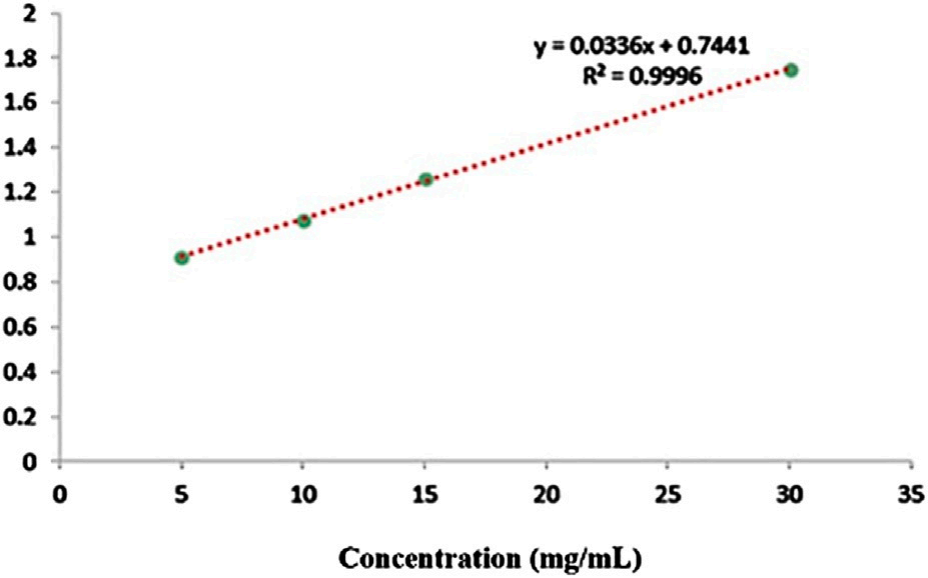
The calibration curve of oligosaccharides by LC-MS.

The SEM image of the oligosaccharide nanoliposome revealed the precise particle size, confirming the DLS results. The average size of the drug-containing nanoliposome observed in the SEM image was approximately 160 nm (Figure 2). The calculated DL was 30.33%, and the EE was 60.67% as related Figure 3 was previously presented.

### 3.2 Polysaccharide release from nanoliposome

**FIGURE 4:**
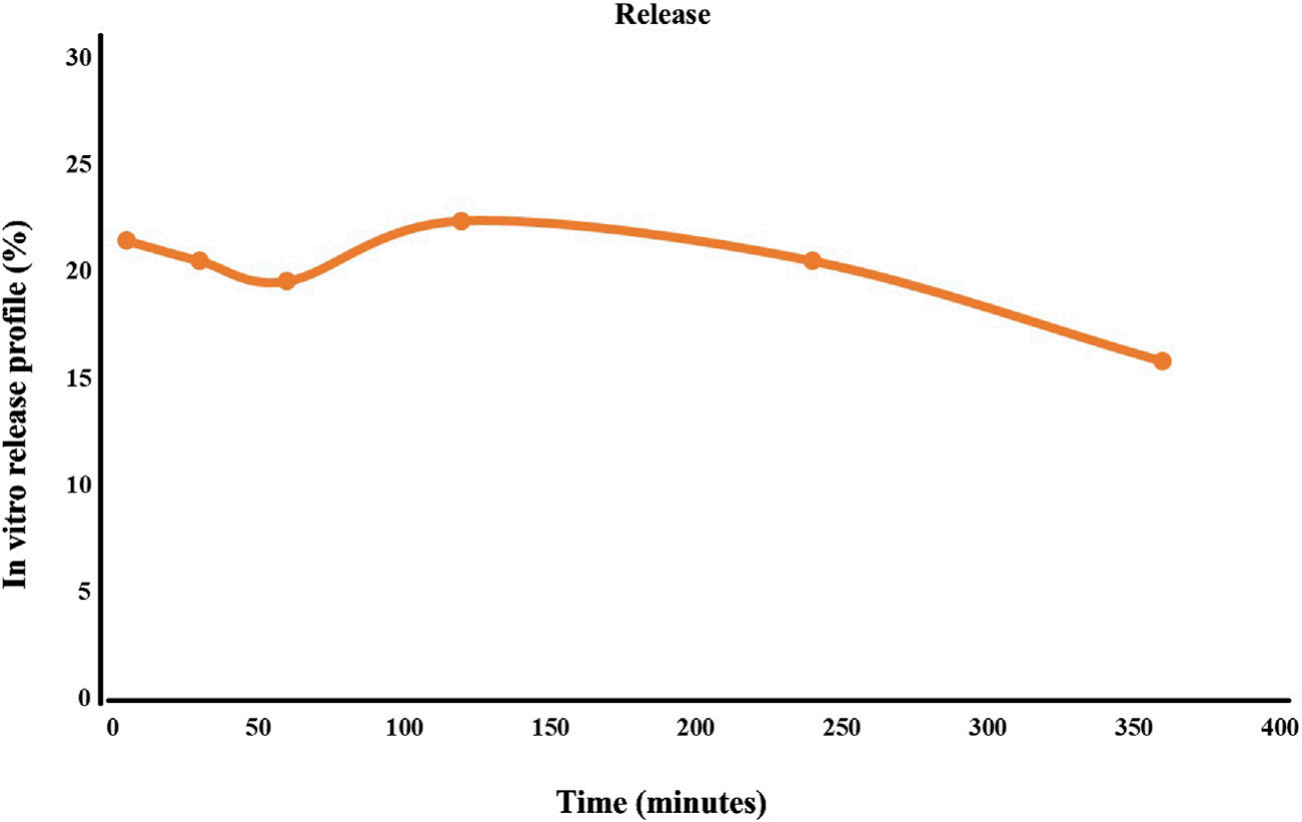
Oligosaccharide release rate from lipid nanocarrier.

The percentage of drug release is presented in Figure 4. Initially, the release curve exhibits a rapid release phase, attributed to the surface-adsorbed oligosaccharides on the nanoliposome. Following this, a steady and controlled release of the polysaccharide was observed. The consistent release over the first four hours could be due to the gradual release of oligosaccharides from the nanoliposomes, coupled with their simultaneous degradation in the surrounding environment. After the four-hour mark, the release of oligosaccharides from the nanoliposomes decreases. At the same time, the degradation continues at the same rate, leading to a reduced concentration of oligosaccharides in the environment and a downward trend in Figure 4.

### 3.3 FT-IR analysis

**FIGURE 5:**
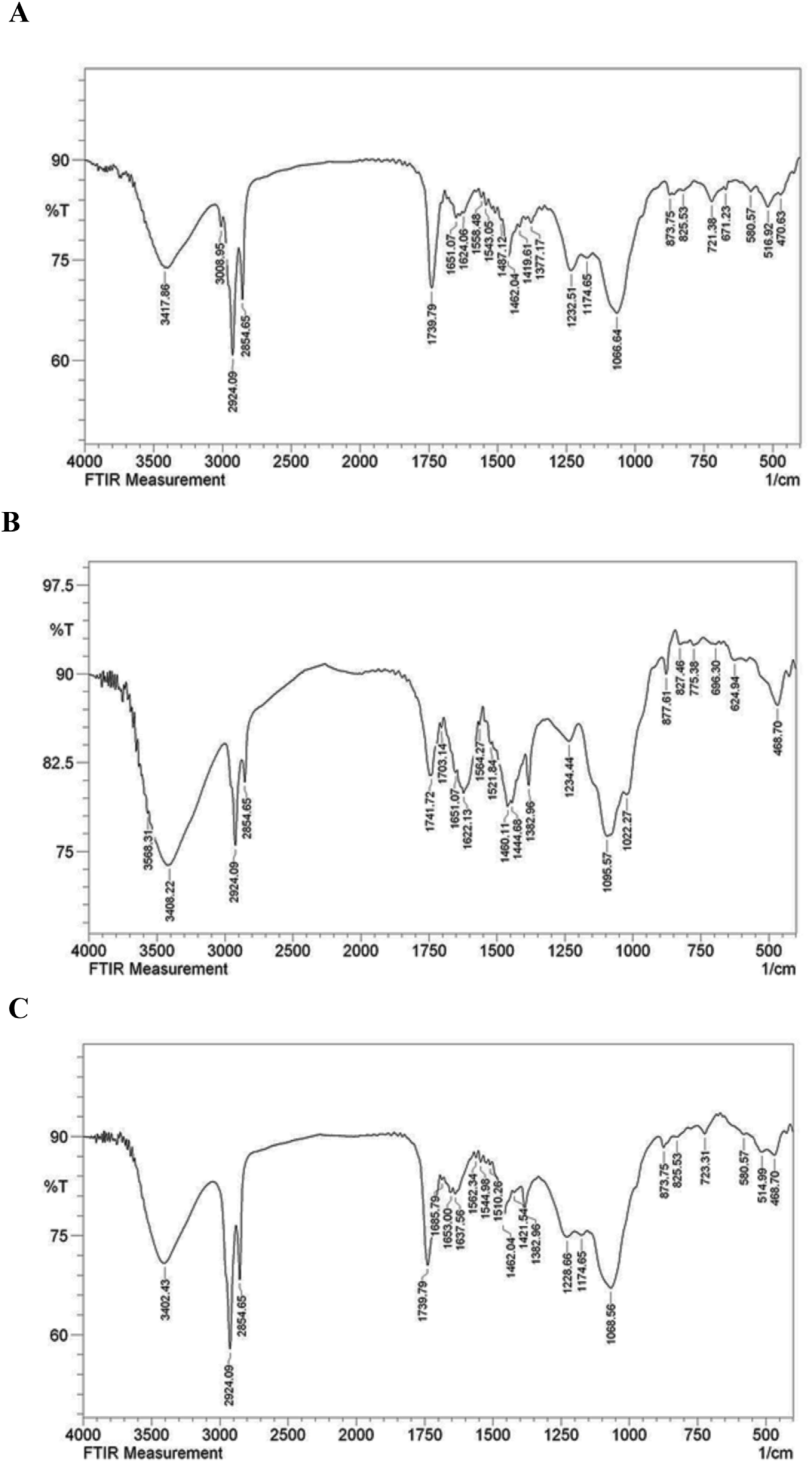
FT-IR spectrum of free drug **(A)**, drug-free liposome **(B)**, Nanoliposome containing drug **(C)**.

The FT-IR spectrum of the nanoliposome containing the oligosaccharides exhibited significant alterations in the bands compared to the spectra of the free oligosaccharides and the drug-free nanoliposome. Notable shifts in sharp bands were observed: from 3,408 to 3,402, 1,651 to 1,653, 1,624 to 1,637, 1,558 to 1,562, 1,419 to 1,421, 1,232 to 1,228, 1,066 to 1,068, 825 to 873, and 470 to 468 (in cm^−1^). These findings confirmed the presence of oligosaccharides in the liposome spectrum. They indicated that the bands in the drug-containing liposome were less intense than those of the free drug and the drug-free liposome. This decrease in intensity is attributed to intermolecular interactions and hydrogen bonding between the CH groups and OH groups of the free drug and the drug-free liposome (Figure 5).

### 3.4 Cytotoxicity assessment

**FIGURE 6:**
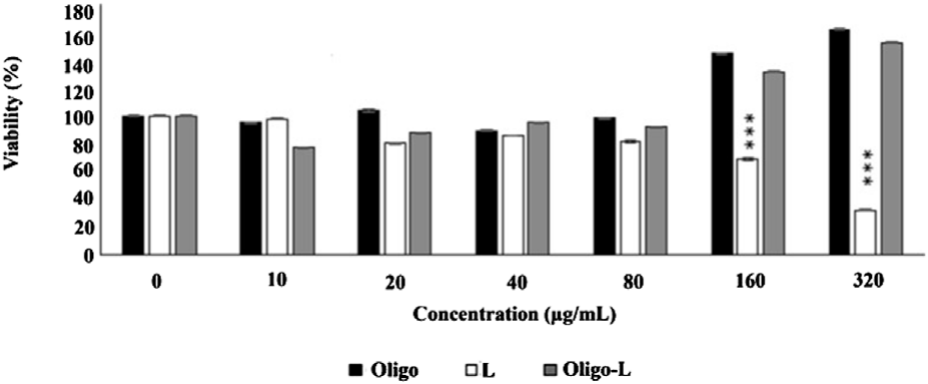
Cytotoxicity in PC12 cells. The data are shown as mean ± S.E.M. ***p < 0.001 vs. Oligo. Oligo, oligosaccharide; L, drug-free liposome; Oligo-L, Nanoliposome containing oligosaccharide.

The cytotoxicity assessment results indicated that increasing the oligosaccharide dose from 10 to 320 μg/mL led to a rise in cell viability from 95.05% to 162.42% after 24 h. Notably, the oligosaccharide derived from *R. canina* at doses ranging from 10 to 320 μg/mL exhibited no significant toxicity. Similarly, elevating the nanoliposome dose containing oligosaccharide from 33 to 1,056 μg/mL (equivalent to 10–320 μg/mL of oligosaccharide) resulted in an increase in cell viability from 77.44% to 152.92% after 24 h. No notable toxicity was observed within the 10–320 μg/mL range of nanoliposome-containing oligosaccharide. Furthermore, drug-free nanoliposomes demonstrated no apparent toxicity up to a concentration of 80 μg/mL, underscoring the biocompatibility of nanoliposomes and their potential for intracellular applications (Figure 6).

### 3.5 Behavioral results

#### 3.5.1 Oligo-L enhanced motor function following SCI

**FIGURE 7:**
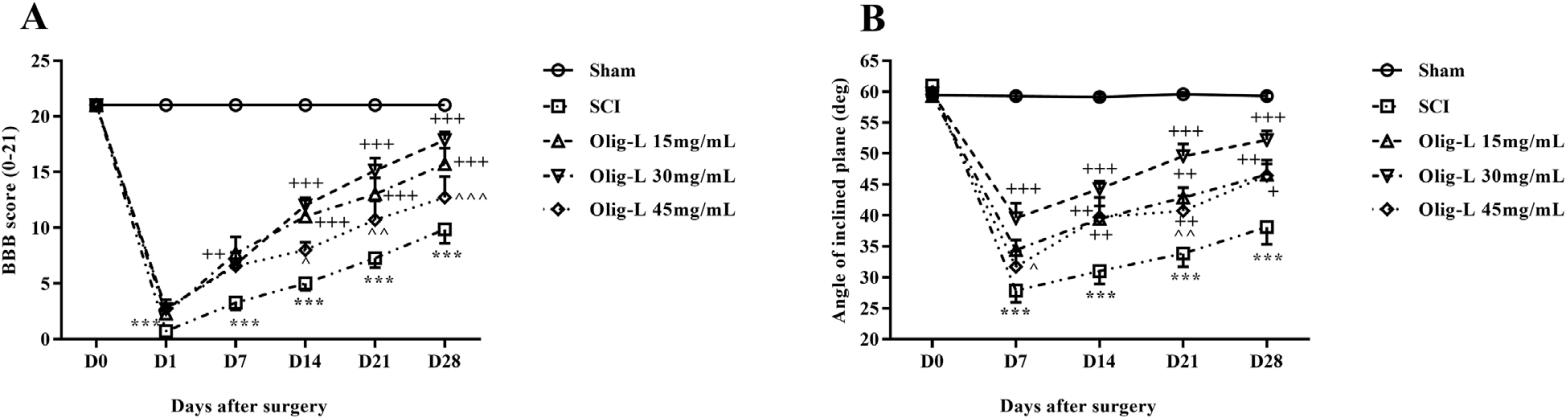
Effects of Oligo-L on locomotor activity after SCI model in rats. BBB score **(A)**, inclined-plane **(B)** test. Data are expressed as mean ± S.E.M. (*n* = 7). A two-way ANOVA was used. ***p < 0.001 vs. sham group and ^+^ *p* < 0.05, ^++^ *p* < 0.01, ^+++^ *p* < 0.001 vs. SCI group and ^^^ *p* < 0.05, ^^^^ *p* < 0.01, ^^^^^ *p* < 0.001 vs. Oligo-L 30 mg/mL group.

The BBB test results indicated that the sham group did not exhibit any movement defects following laminectomy, and all rats scored 21 throughout the treatment period. In contrast, the motor ability of the SCI group significantly decreased compared to the sham group (*p* < 0.001). Treatment with various doses, especially the dose of 30 mg/mL Oligo-L, led to a noticeable enhancement in motor function, which was evident from the first week (*p* < 0.05) (Figure 7A).

The results of the inclined plane test showed that the animals in the Sham group did not have any movement impairments after the laminectomy and could maintain their balance on the plane at angles up to 60°. On the other hand, the SCI group showed a significant decrease in their ability to maintain balance on the inclined surface, and this decrease persisted throughout the treatment period compared to the Sham group (*p* < 0.001). The administration of various doses of oligosaccharide nanoformulation increased the maximum balance angle on the inclined plane starting from the seventh day after the injury (*p* < 0.05). The most noticeable improvement was observed in the Oligo-L 30 mg/mL group compared to the other treatment groups (*p* < 0.05) (Figure 7B).

#### 3.5.2 Oligo-L reduced neuropathic pain following SCI

**FIGURE 8:**
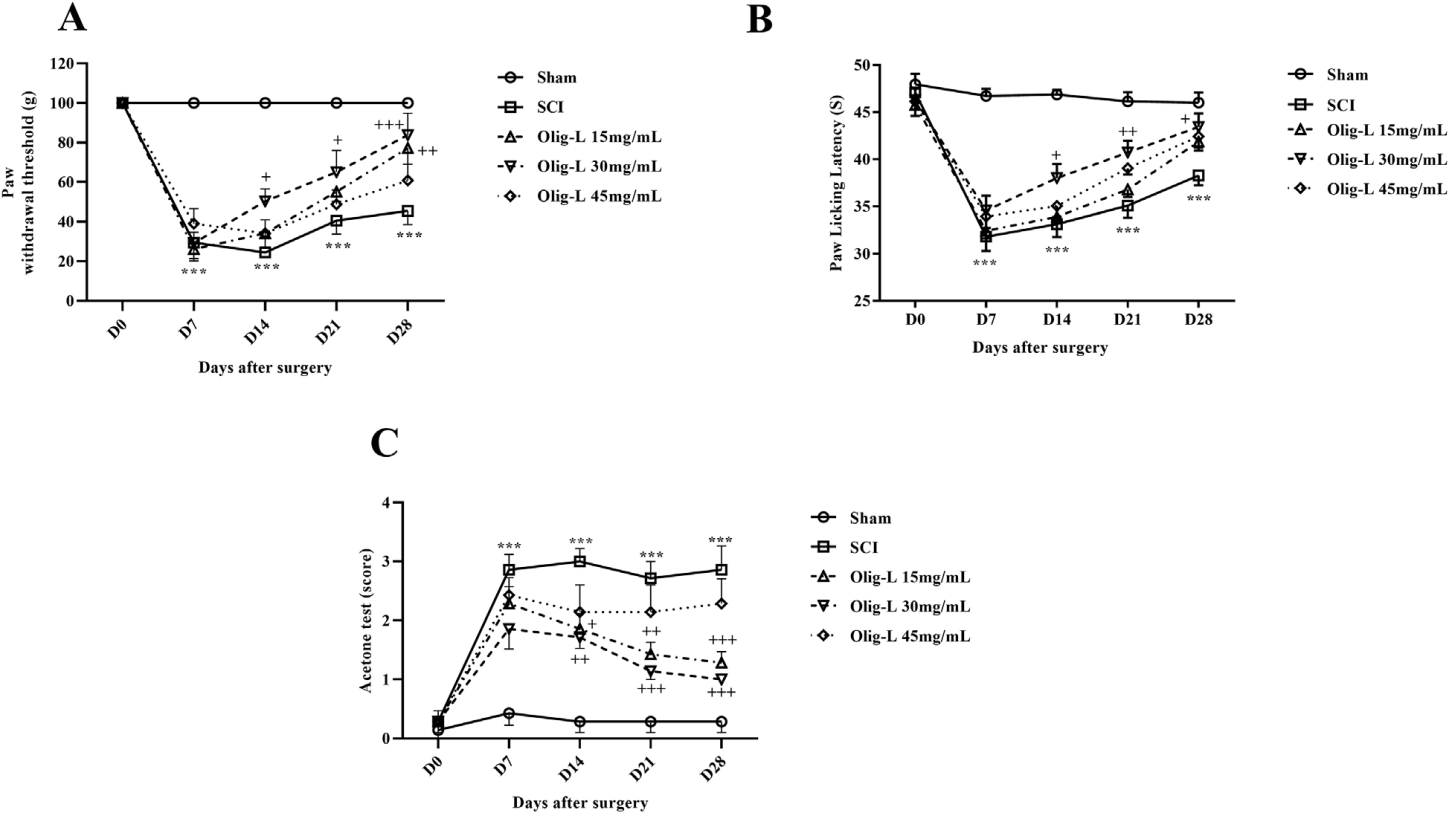
Effects of Oligo-L on pain after SCI model in rats. Mechanical pain **(A)**, thermal pain **(B)**, and cold pain **(C)**. Data are expressed as mean ± S.E.M. (*n* = 7). A two-way ANOVA was used. ***p < 0.001 vs. sham group and ^+^ *p* < 0.05, ^++^ *p* < 0.01, ^+++^ *p* < 0.001 vs. SCI group.

The von Frey test results showed that the mechanical pain threshold in the Sham group remained constant over the 28 days after laminectomy. In contrast, the SCI group demonstrated a significant decrease in pain tolerance threshold, with the animals experiencing pain (*p* < 0.001). Treatment with different doses of Oligo-L increased pain threshold in rats (*p* < 0.05). The recovery process was especially significant in animals receiving the Oligo-L 30 mg/mL compared to the other groups (Figure 8A).

The hot plate test results indicated that laminectomy did not impact the response threshold of rats in the Sham group to the thermal stimulus. However, compressive injury led to a notable decrease in thermal pain threshold tolerance and increased pain sensation in the animals (*p* < 0.001). Treatment with a 30 mg/mL dose of Oligo-L formulation demonstrated effective reduction of thermal pain and enhancement of pain tolerance threshold (*p* < 0.05) (Figure 8B).

The results of the cold tolerance test showed that after laminectomy, rats did not react to acetone spray and this stimulus was not painful for them. However, the animals in the SCI group showed a strong reflex to acetone and sensed cold pain (*p* < 0.001). Treatment with various doses of Oligo-L, especially dose 30 mg/mL, was associated with an increase in the cold pain tolerance threshold from the 7th day after the injury (*p* < 0.05) (Figure 8C).

#### 3.5.3 Oligo-L restored weight changes following SCI

**FIGURE 9:**
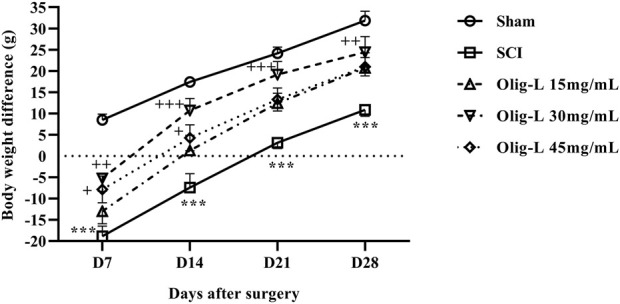
Effects of Oligo-L on body weight changes after SCI model in rats. Data are expressed as mean ± S.E.M. (*n* = 7). A two-way ANOVA was used. ***p < 0.001 vs. sham group and ^+^ *p* < 0.05, ^++^ *p* < 0.01, ^+++^ *p* < 0.001 vs. SCI group.

The rats’ weight changes were calculated as a percentage relative to day 0. Over 4 weeks, the weight of the animals in the Sham group increased, while the rate of weight gain in the SCI groups was slower compared to the Sham group (*p* < 0.001). Treatment with various doses of the Oligo-L formulation reversed the weight loss after SCI and resulted in significant weight gain (*p* < 0.01). The most significant weight gain was observed in the group receiving the Oligo-L 30 mg/mL (Figure 9).

### 3.6 Biochemical result

#### 3.6.1 Oligo-L reduced oxidative stress following SCI

**FIGURE 10:**
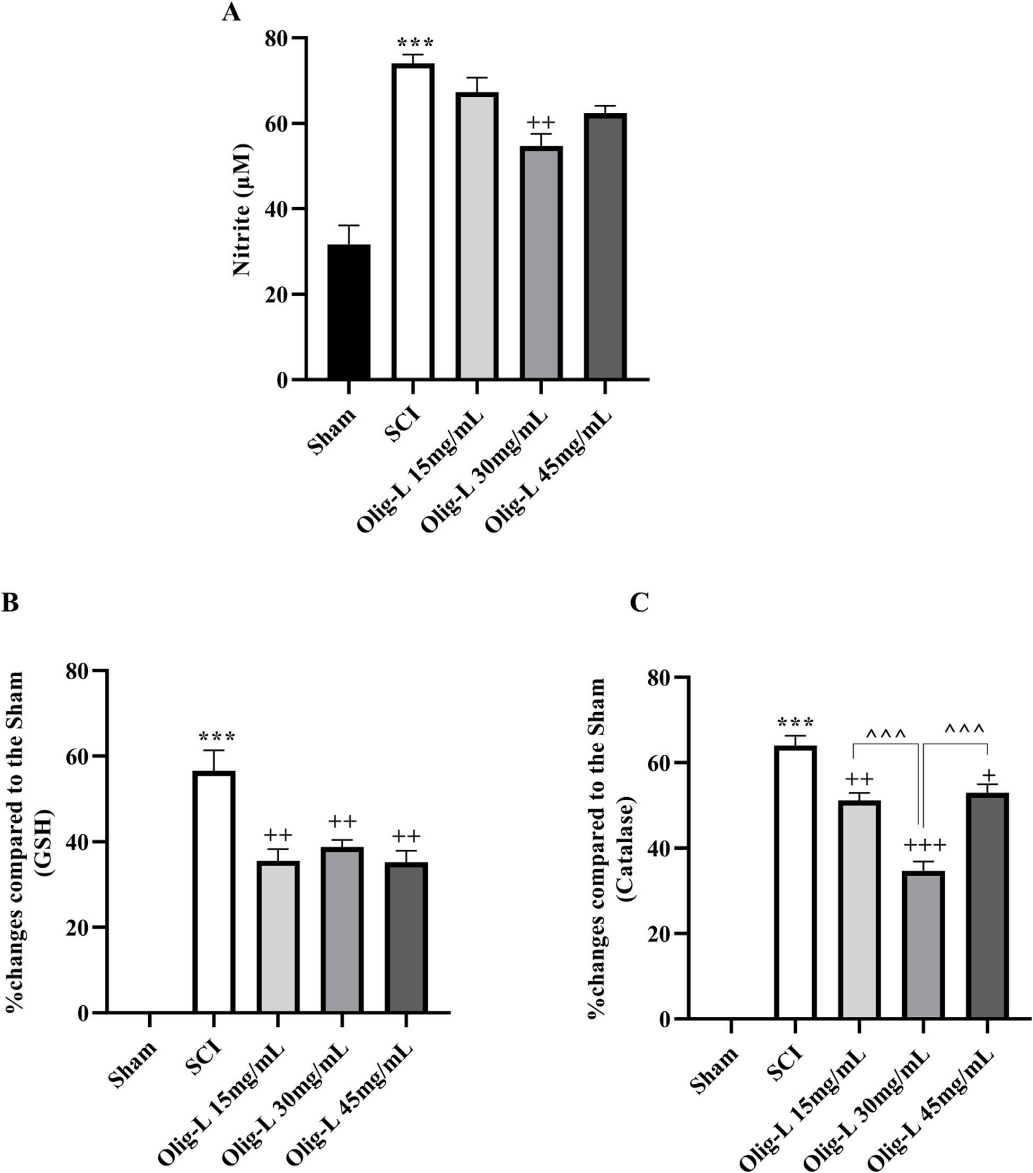
Effects of Oligo-L on oxidative stress factors after SCI model in rats. Nitrite **(A)**, glutathione **(B)** and catalase **(C)**. Data are expressed as mean ± S.E.M. (*n* = 3). One-way ANOVA was used. ***p < 0.001 vs. sham group and ^+^ *p* < 0.05, ^++^ *p* < 0.01, ^+++^ *p* < 0.001 vs. SCI group and ^^^^^ *p* < 0.001 vs. Oligo-L 30 mg/mL group.

Compared to the sham group, the SCI group showed a notable increase in serum nitrite levels (*p* < 0.001). Treatment with three doses of Oligo-L led to a reduction in serum nitrite levels compared to the SCI group. These decreasing changes were particularly significant in the Oligo-L 30 mg/mL group (*p* < 0.05, Figure 10A). The findings revealed that the SCI group had significantly lower levels of serum GSH and catalase compared to the sham group (*p* < 0.001). However, treatment with Oligo-L, especially at the Oligo-L 30 mg/mL, effectively reversed this decrease (*p* < 0.05) (Figures 10B, C).

#### 3.6.2 Oligo-L preserved neurons following SCI

**FIGURE 11:**
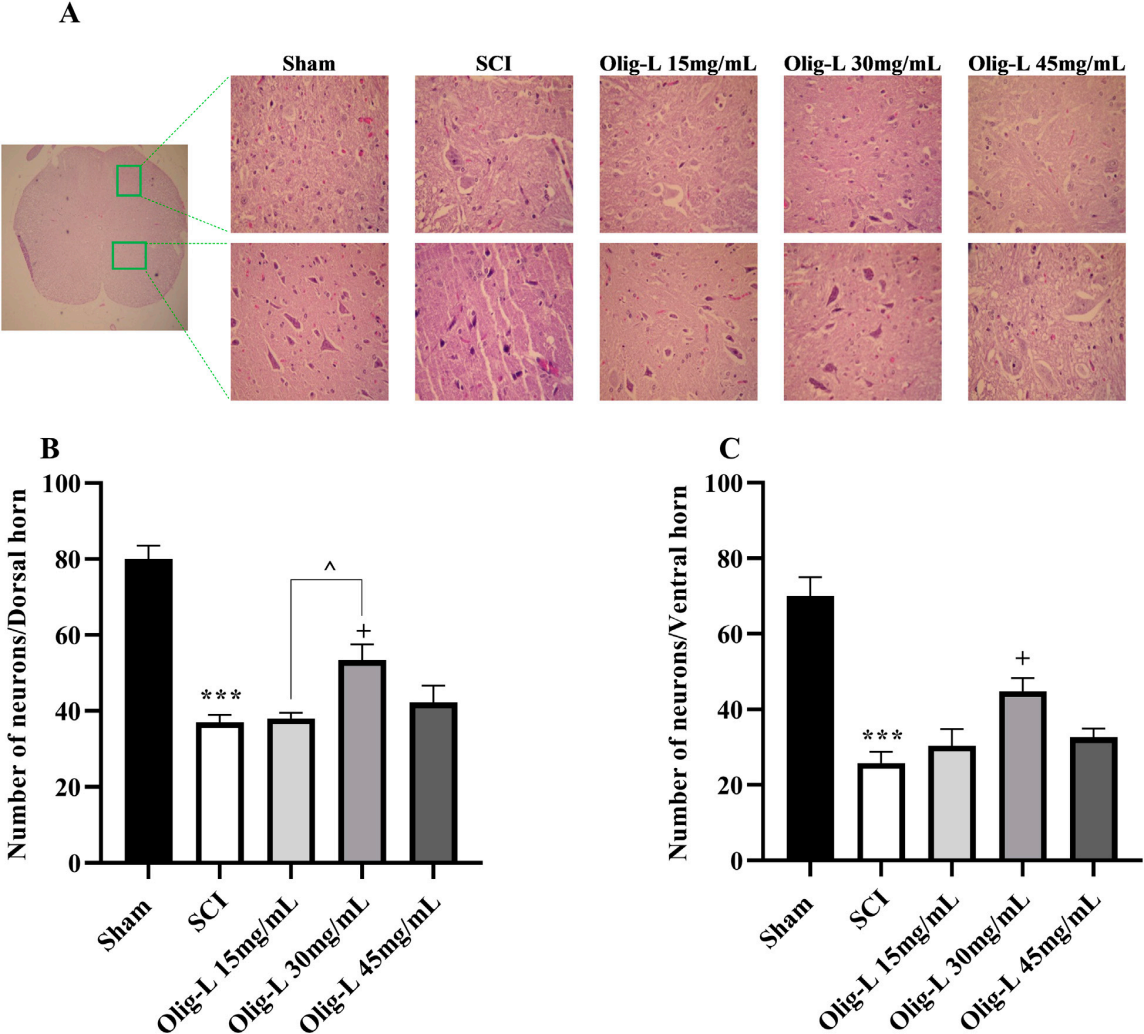
Effects of Oligo-L on the number of preserved neurons after SCI model in rats. Sensory neurons in the dorsal horn **(A)** and motor neurons in the ventral horn **(B)**. Data are expressed as mean ± S.E.M. (*n* = 3). One-way ANOVA was used. ^**^ *p* < 0.01, ***p < 0.001 vs. sham group and ^+^ *p* < 0.05 vs. SCI group and ^^^ *p* < 0.05 vs. Oligo-L 30 mg/mL group.

Quantifying the motor neurons in the ventral horn and sensory neurons in the dorsal horn of the spinal cord’s gray matter showed a substantial reduction in neuron count at the lesion site following SCI compared to the Sham group (*p* < 0.01 and *p* < 0.001, respectively) (Figure 11). Furthermore, treatment with Oligo-L at a concentration of 30 mg/mL preserved more neuron numbers in both the dorsal and ventral regions than other treatment doses (*p* < 0.05).

## 4 Discussion

This study extensively investigated the physical properties of a nanoliposomal formulation containing oligosaccharides extracted from *R. canina* and assessed their effectiveness in improving motor function, reducing neuropathic pain, combating oxidative stress, and increasing neuronal survival in rats with SCI. The compression model was used to induce SCI, resulting in sensory and motor impairments in the rats. Various tests, including acetone drop, von Frey, hot plate, BBB, and inclined plane tests, were conducted to evaluate these impairments. Overall, the administration of the nanoliposomal formulation significantly improved sensory and motor deficits in the rats and helped counteract weight loss induced by SCI, leading to significant weight gain. The protective effects of the Oligo-L were linked to their impact on oxidative stress parameters. Histological analysis showed that the Oligo-L increased the number of sensory and motor neurons in the posterior and ventral horns of the spinal cord.

SCI is a debilitating neurological condition that results in significant impairments in movement, sensation, and autonomic function. The pathophysiology of SCI involves both acute and chronic stages, characterized by a cascade of destructive processes including ischemia, oxidative stress, inflammation, apoptosis, and subsequent disorders (Guest et al., 2022). Among various models of SCI, the spinal cord compression model closely mimics the pathological features observed in human injuries (Poon et al., 2007). Therefore, in this study, we used the compression SCI model to investigate these pathological changes. Depending on the location and severity of the injury SCI can have profound effects on motor and sensory function (Motiei-Langroudi and Sadeghian, 2017). In this study, we induced complete SCI in the 8th and 9th thoracic vertebrae, which resulted in complete paralysis of the animal and loss of sensation in rats.

*Rosa canina*, often referred to as dog rose, is a multifaceted medicinal plant with a deep-rooted history in traditional medicine. Its notable anti-inflammatory, antioxidant, and immunomodulatory effects rendered *R. canina* a highly valuable component in herbal remedies and dietary supplements. It boasts a wealth of bioactive compounds and nutrients that enhance its therapeutic properties (Khazaei et al., 2020). Among the active compounds of *R. canina*, oligosaccharides, carbohydrate molecules composed of a small number of linked monosaccharide units, exhibited significant anti-diabetic properties by influencing gene expression and DNA methylation (Javid et al., 2024). We have previously characterized the chemical structure of the aforementioned oligosaccharide (Rahimi et al., 2020). Isolated polysaccharides extracted from *R. canina* enhanced Notch signaling and cyclin D1 expression, and improved cellular function (Sajadimajd et al., 2022). Also, the protective effects of *R. canina* have been observed via autophagy enhancement (Sajadimajd et al., 2020). In addition to animal studies, a randomized, double-blind, placebo-controlled clinical trial confirmed the safety and efficacy of a polysaccharide extracted from *R. canina* in patients with non-alcoholic fatty liver disease (Bahrami et al., 2022).

Nanoliposomes are small lipid-based vesicles that can encapsulate therapeutic agents, enhancing their delivery to targeted sites, such as the injured spinal cord. Their unique properties, including biocompatibility, low toxicity, and the ability to penetrate the blood-spinal cord barrier (BSCB), make them promising candidates for SCI treatment (Chakraborty et al., 2021; Lu et al., 2023). In the present study, nanoliposomes containing oligosaccharides extracted from *R. canina* with a size of 188.0 nm, a zeta potential of −18.5 mV, and 60.67% were prepared. Our results indicated that Oligo-L formulation improved the movement disorder in animals with SCI. Specifically, the injection of 10 µL of a dose containing 30 mg/mL of the Oligo-L formulation led to improvements in the animals’ movements and functional characteristics. According to a report, nanoparticles containing prostaglandin E1 significantly enhanced the recovery from hind-limb motor dysfunction caused by SCI in rats. The continuous release of prostaglandin E1 from the nanoparticles is believed to have supported cell survival and natural repair processes, ultimately resulting in improved functional recovery (Takenaga et al., 2010). Wu et al. demonstrated that nanoparticles containing ferulic acid-modified glycol chitosan exhibited a notable impact on enhancing motor function following SCI (Wu et al., 2014). An et al. research findings showed that SCI rats treated with nanoliposomes containing minocycline hydrochloride and dextran sulfate gave elevated behavioral scores (An et al., 2022). In another study, it was demonstrated that the conjugation of adenosine with lipid squalene to form nanoparticles allows for prolonged circulation of this nucleoside, and was associated with neuroprotection in a SCI model. Animals treated with these nanoparticles displayed early improvement in hind-limb motor function (Gaudin et al., 2014). Also reported that nanoformulated curcumin improved motor activity after SCI in rats (Krupa et al., 2019).

Neuropathic pain is a prevalent and incapacitating consequence of SCI, affecting as many as 80% of patients. The underlying pathophysiology encompasses a series of structural, neurochemical, and inflammatory alterations in the central nervous system, leading to neuronal hyperexcitability, and disruption and imbalance of pain pathways (Lee et al., 2013; Forte et al., 2022). In the present study, neuropathic pain resulting from SCI significantly improved following administration of Oligo-L extracted from *R. canina*. In this study, the average dose of Oligo-L formulation led to an increase in the pain tolerance threshold in all three types of mechanical, thermal, and cold pain compared to the SCI group. A clinical trial found that the powdered extract from *R. canina* showed mild to moderate efficacy in reducing pain among osteoarthritis patients (Christensen et al., 2008). A study showed that Muscovite nanoparticles alleviated mechanical and cold allodynia in the neuropathic pain model as assessed by the von Frey and acetone tests (Oh et al., 2020). In a model of peripheral neuropathic pain, researchers observed that cerium oxide nanoparticles successfully alleviated mechanical allodynia, cold allodynia, and thermal hyperalgesia in rats (Forouzanfar et al., 2023). It was reported that long-term administration of nanocurcumin can improve neuropathic pain-related behavior (Saffarpour et al., 2021).

Weight loss is a common outcome of SCI. The early phases of SCI are marked by a reduction in energy expenditure and an elevated catabolic rate, which can persist for weeks to months (Kearns et al., 1992; Powell et al., 2017). In our study, the administration of an Oligo-L formulation counteracted weight loss post-SCI and led to notable weight gain alterations. Previously, Powell et al. reported that nanoparticles containing antioxidant enzymes enhanced the locomotor performance and weight gain of rats following SCI (Andrabi et al., 2020).

Following SCI, the primary trauma sets off a series of events that worsen the damage, primarily through oxidative stress (Jia et al., 2012). The spinal cord tissue with its high lipid content and metabolic activity, is particularly vulnerable to oxidative damage. Post-injury, there is an increase in ROS can overwhelm the body’s natural antioxidant defenses, leading to mitochondrial dysfunction and neuronal cell death. This oxidative stress is recognized as a key feature of the secondary injury phase, which can manifest hours to weeks after the initial trauma (Jia et al., 2012; Visavadiya et al., 2016). Daels-Rakotoarison et al. showed that the *R. canina* extract successfully suppressed the generation of ROS (Daels-Rakotoarison et al., 2002). NO is generated in two distinct phases following SCI. The initial phase involves the upregulation of neuronal nitric oxide synthase (nNOS) in spinal cord cells, leading to a rapid surge in NO levels. Then, a secondary phase emerges, characterized by the activation of inducible nitric oxide synthase (iNOS) in inflammatory cells, resulting in sustained elevated levels of NO (Conti et al., 2007). Elevated NO concentrations can combine with superoxide radicals to produce peroxynitrite (ONOO^−^), a potent oxidant that exacerbates oxidative stress and cellular damage. Peroxynitrite has the potential to trigger lipid peroxidation, disrupt mitochondrial function, and set off a chain of adverse effects, including excitotoxicity and inflammatory responses (Liu et al., 2000; Yin et al., 2024). Furthermore, the heightened levels of ROS and NO initiate kinase signaling cascades and the phosphoinositide 3-kinase (PI3K) pathway. This interaction can amplify downstream signaling effects, impacting processes of neuroinflammation and apoptosis (Zhang et al., 2016). Our results showed that the use of oligosaccharide nanoformulation extracted from *R. canina* was able to reduce the increased amount of nitric oxide following SCI. Andrabi et al. found that nanoparticles containing antioxidant enzymes preserved the injured spinal cord from apoptosis by mitigating mitochondrial dysfunction (Andrabi et al., 2020).

GSH, a non-enzymatic antioxidant, scavenges reactive oxygen and nitrogen species and is a substrate for detoxification enzymes like glutathione peroxidase and glutathione reductase (Vašková et al., 2023). Research indicated that the depletion of glutathione amplifies various secondary pathways that play a role in motor neuron degeneration following SCI (Visavadiya et al., 2016; Stewart et al., 2022). On the other hand, catalase is a crucial antioxidant enzyme that by the oxidation of NO, decreases its bioavailability (Brunelli et al., 2001). Our results demonstrated that the use of Oligo-L can effectively restore the diminished levels of glutathione and catalase after SCI. The nanoliposomes with resveratrol and puerarin decreased the production of free radicals and increased the levels of antioxidants GSH and catalase following reperfusion injury in rats, which effectively reduced oxidative stress (Chen et al., 2020). In addition to these protections and improvement of sensory and motor function, this nanoliposome was also effective in preserving spinal cord tissue and sensory and motor neurons. A study on animals revealed that the aqueous extract of *R. canina* enhanced neuronal density in regions of the hippocampus in mice (Amintaheri et al., 2017).

## 5 Conclusion

In summary, our findings highlighted the potential of *R. canina* oligosaccharide nanoliposomal formulation as a promising therapeutic strategy for reducing the debilitating effects of SCI. By addressing the critical role of oxidative stress in the pathophysiology and development of SCI, this approach not only improved motor function and reduced neuropathic pain but also demonstrated a neuroprotective effect on neuronal survival. As SCI is a complex public health concern with limited therapeutic options, the results of our study emphasized the importance of investigating natural compounds and advanced delivery systems for the development of innovative interventions.

## Data Availability

The raw data supporting the conclusions of this article will be made available by the authors, without undue reservation.

## Glossary

BBB: Basso-Beattie-Bresnahan
BSCB: blood-spinal cord barrier
**CO**_**2**_: carbon dioxide
DL%: drug loading
DLS: dynamic light scattering
DNA: Deoxyribonucleic Acid
DTNB: 5,5′-dithiobis (2-nitrobenzoic acid)
EE%: entrapment efficiency
FDA: U.S. Food and Drug Administration
FTIR: Fourier-transform infrared spectroscopy
LC-MS: liquid chromatography-mass spectrometry
GSH: glutathione
GSSG: glutathione oxidized
H&E: hematoxylin and eosin
**H**_**2**_ **O**_**2**_: hydrogen peroxide
iNOS: inducible nitric oxide synthase
i.t.: intrathecal
KUMS: Kermanshah University of Medical Sciences
NIH: National Institutes of Health
nNOS: neuronal nitric oxide synthase
NO: nitric oxide
MTT: 3-[4,5-dimethylthiazol-2-yl]-2,5 diphenyl tetrazolium bromide
Oligo-L: Rosa canina oligosaccharide liposomal formulation
ONOO−: peroxynitrite
PDI: polydispersity index
PI3K: phosphoinositide 3-kinase
ROS: reactive oxygen species
SCI: spinal cord injury
UV–vis: ultraviolet-visible spectroscopy
**W**_**t**_: initial amount of the drug
**W**_**f**_: amount of free drug in the supernatant solution
**W**_**s**_: total amount of the nanoliposomes
